# Nursing effect of ECMO combined with CRRT in the treatment of fulminant myocarditis: A protocol of randomized controlled trial

**DOI:** 10.1097/MD.0000000000024085

**Published:** 2021-01-22

**Authors:** Juan Wu, Hui Zhang, Yongxia Gao, Xihua Huang

**Affiliations:** Department of Emergency Intensvie Care Unit, The First Affiliated Hospital With Nanjing Medical University, Nanjing, Jiangsu Province, China.

**Keywords:** combination therapy, continuous renal replacement therapy, extracorporeal membrane oxygenation, fulminant myocarditis, randomized controlled trial

## Abstract

**Background:**

Fulminant myocarditis has a sudden onset and rapid progress, which can easily cause multiple organ failure. Acute kidney injury is a common complication. ECMO (extracorporeal membrane oxygenation) and CRRT (continuous renal replacement therapy) have been used in the treatment of fulminant myocarditis, but the combination of the 2 has an impact on the prognosis. There is still a big controversy. Therefore, the purpose of this randomized controlled trial is to evaluate the nursing effect and long-term efficacy and safety of ECMO combined with CRRT in the treatment of fulminant myocarditis.

**Methods:**

This is a prospective randomized controlled trial to study the effectiveness and safety of ECMO combined with CRRT in the treatment of fulminant myocarditis. Approved by the clinical research ethics committee of our hospital. Patients were randomly assigned to 1 of 2 treatment options:

1. ECMO combined with CRRT group and
2. ECMO group.

Observation indicators include: basic vital signs, laboratory indicators, echocardiographic changes, complications, and outcomes. SPSS 25.0 (Chicago, IL) version statistical software package was used to analyze the data.

**Discussion:**

This study will evaluate the nursing effect and long-term efficacy and safety of ECMO combined with CRRT in the treatment of fulminant myocarditis. The results of this experiment will provide clinical evidence for the treatment of fulminant myocarditis with ECMO and CRRT.

**Ethics and dissemination:**

Private information from individuals will not be published. This systematic review also does not involve endangering participant rights. Ethical approval was not required. The results may be published in a peer-reviewed journal or disseminated at relevant conferences.

**OSF Registration number:**

DOI 10.17605/OSF.IO/PAQBZ.

## 1 Introduction

Fulminant myocarditis is a systemic disease dominated by myocardial inflammatory damage caused by infections, autoimmune diseases, etc. Patients can quickly develop severe hemodynamic disturbances, heart failure, and severe malignant arrhythmia, leading to multiple organs such as liver and kidney dysfunction, the early mortality rate is extremely high.^[1,2]^ Early adoption of “A life support-based comprehensive treatment regimen” including mechanical life support, immunomodulatory therapy, antiviral therapy (especially neuraminidase inhibitors), reduce the hospital mortality rate of FM patients.^[3]^

Extracorporeal membrane oxygenation (ECMO) takes the venous blood in the body out of the body and injects it into the patient's arteries or veins through a special material artificial heart-lung bypass oxygenation device, which plays a role of partial heart-lung replacement.^[4]^ It is believed that ECMO can be used as a first-line treatment option for fulminant myocarditis.^[5]^ Fulminant myocarditis often causes renal hypoperfusion, and in ECMO circulation support, hemolysis caused by red blood cell destruction can cause acute kidney injury (AKI), which seriously affects the prognosis of patients.^[6]^ Studies have shown that AKI and overload fluid occur in 70% to 85% of ECMO patients.^[7]^ Continuous renal replacement therapy (CRRT) is an extracorporeal blood purification therapy that can replace impaired renal function for a long time. It removes toxins and cytokines through diffusion and convection methods, and reduces cardiac load through ultrafiltration Maintaining hemodynamic stability well.

The impact of ECMO combined with CRRT on the prognosis is still controversial. Studies have pointed out that regardless of whether AKI occurs, and whether CRRT is performed, the difference in mortality of CF patients in the intensive care unit is not statistically significant.^[8]^ Therefore, this research program will study the effectiveness and safety of ECMO combined with CRRT in the treatment of fulminant myocarditis through randomized controlled trials.

## 2 Materials and methods

### 2.1 Study design

**Figure 1:**
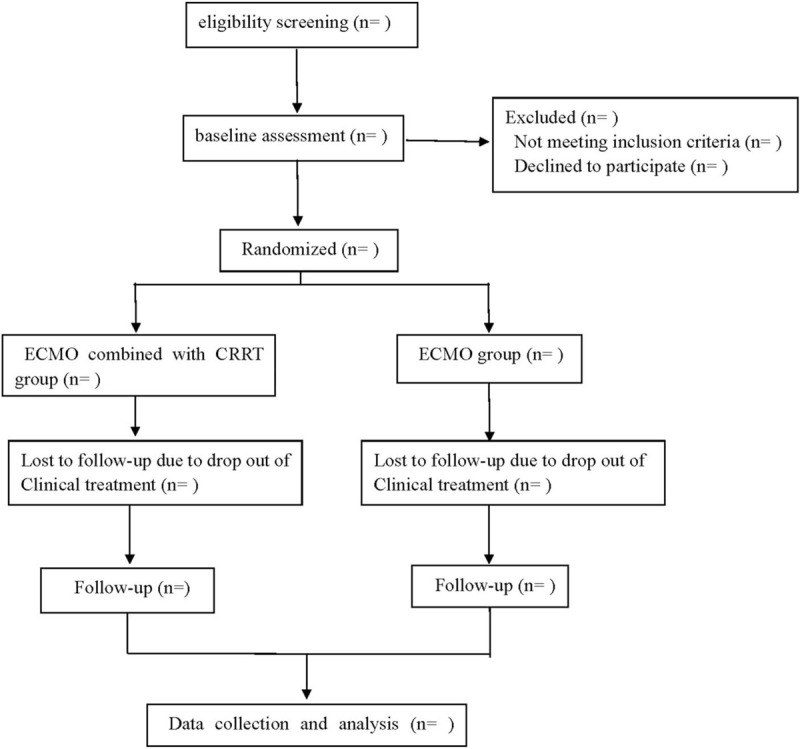
Flow diagram.

This is a prospective randomized controlled trial to study the effectiveness and safety of ECMO combined with CRRT in the treatment of fulminant myocarditis. We followed the Consolidated Standards of Reporting Trials (CONSORT) guidelines for reporting randomized trials and provided a CONSORT flow diagram (Fig. 1) and the Standard Protocol Items: Recommendations for Interventional Trials (SPIRIT) 2013 statement.

### 2.2 Ethics and registration

This research program is in accordance with the Helsinki Declaration and approved by the Clinical Research Ethics Committee of our hospital. This protocol has been registered in open Science Framework (OSF)(Registration number: DOI 10.17605/OSF.IO/PAQBZ). All patients need to sign a written informed consent before they are randomly assigned to continue the trial.

### 2.3 Sample size

In order to draw reliable conclusions, this study will include as many qualified samples as possible, referring to similar studies,^[8]^ we set the minimum number of people in each group of studies to 40, and the final number will be based on actual recruitment.

### 2.4 Patients

Inclusion criteria: ①Meet the diagnostic criteria for fulminant myocarditis of the European Heart Collaboration Group;^[9]^②No previous serious underlying heart disease; ③A viral infection of the respiratory system occurred before the onset; ④After the onset, the condition deteriorated rapidly, the left heart function was severely impaired, the hemodynamics was unstable, and circulatory and respiratory support was needed; ⑤Be over 18 years old.

Exclusion criteria: ①Those who use ECMO for other reasons; ②;Severe irreversible multiple organ damage; ③Pregnant women with severe heart failure; ④Patients with congenital and acquired diseases that cannot be corrected after cardiac surgery; ⑤Cardiopulmonary Resuscitation (CPR) for more than 30 minutes; ⑥Thouse who are unable to understand the research plan after explanation or unwilling to participate.

### 2.5 Study design

Eligible participants were randomly assigned to the treatment group or the control group at a ratio of 1:1 using a random tool based on the central network. Randomization was performed without any stratification generated by an independent statistician who was not involved in trial implementation or statistical analysis using SAS 9.3 software (SAS Institute, Cary, NC). The clinical research coordinator entered the participant information on the tablet, and was given a random number. The research assistant gets the participant's assignment from the computer. Throughout the research process, the research assistant is responsible for screening, recruiting participants, and assigning random numbers to the included participants. The result assessor is responsible for the assessment of the scale.

### 2.6 Interventions

1. Establishment of ECMO: The surfaces of all connecting pipes of the ECMO system, the contact surfaces of the oxygenator and the centrifugal pump are all covered with heparin coating. After ultrasound assessment of blood vessel diameter and elastic plaque, the femoral vein (20–22 Fr) and femoral artery (16–20 Fr) were inserted percutaneously. If severe ischemia occurs in the lower extremities, place a 6F perfusion tube to increase blood supply to the lower limbs. The perfusion tube increases blood supply to the lower extremities. The femoral vein femoral artery catheterization was used to establish an ECMO (Veno-arterial Extracorporeal Membrane Oxygenation) support model.
2. ECMO management: ①Adjust pump flow according to cardiac function and hemodynamic parameters; ②Anticoagulation management: use unfractionated heparin for anticoagulation, monitor the activated coagulation time every 2 hours, and adjust the amount of heparin to maintain it between 160 and 200 seconds; ③Mechanical ventilation management: mechanically assisted ventilation according to protective lung ventilation strategies; ④Hemodynamic management: monitor heart rate, blood pressure of right upper limb, blood oxygen saturation of right hand. Measure blood gas and central venous oxygen saturation at 4 hour intervals; ⑤Management of vasoactive drugs: adjust the type and dosage of vasoactive drugs according to circulatory indicators such as blood pressure and heart rate.
3. CRRT establishment: In ECMO combined with CRRT treatment, if the 2 loops are placed separately, it will inevitably increase the risk of bleeding and infection, and there are problems such as expensive and complicated operations.^[10]^ The current conventional method is to add the CRRT machine to the ECMO loop, which can avoid the risk of multiple tube placement. When ECMO is combined with CRRT treatment, the connection method of the pipeline is to join the CRRT loop in parallel on the ECMO pipeline. The inlet end (artery) of CRRT is connected to the back end of the centrifugal pump through a three-way connection, and the outlet end (venous) is connected to the other end is before the membrane lung.^[11]^ Choose continuous venous-venous hemofiltration or diafiltration mode, the replacement dosage is pre-dilution (or dialysis) 1 to 2 L/hour, post-dilution 1 L/hour, replacement fluid and dialysate use modified port formula replacement fluid. The nurse adjusts the pump speed according to the doctor's advice according to the acid-base level of the blood gas analysis; adjusts the potassium chloride dosage according to the blood potassium level. Soak and flush the pipeline with 0.9% sodium chloride solution. No additional anticoagulant measures are added during CRRT.
4. ECMO weaning indication:^[12]^ ECMO assisted flow rate is reduced to 1 L/min, no or only small doses of vasoactive drugs are used to maintain hemodynamic stability, mean arterial pressure >60 mm Hg (1 mm Hg = 0.133 kPa), Pulse pressure difference >20 mm Hg, LVEF(left ventricular ejection fraction)>35% to 40%, central venous pressure <10 mm Hg, left ventricular pressure <12 mm Hg, systemic central venous oxygen saturation >65% to 70%, lactic acid <2 mmol/L.
5. Nursing intervention: The operation of the machine and the setting of parameters should be closely monitored in nursing. The nurse observes and records the speed and flow rate of the centrifugal pump every hour, and observes the pressure before and after the pump to avoid hemolysis caused by excessive negative pressure; observe the pressure before and after the oxygenator, observe whether the pipeline shakes, the color of the pipeline, and whether the flow rate is suddenly high suddenly low to prevent insufficient blood flow, thrombosis or pericardial tamponade, etc.; check the temperature setting of the water tank and actual water temperature, etc.; closely monitor heart rate, heart rhythm, blood pressure, urine output, bedside electrocardiogram, left ventricular systolic function and change trends; use blood purification A dedicated observation record sheet, which accurately records data such as arterial pressure, venous pressure, filtrate pressure measurement, transmembrane pressure, pressure drop, fluid flow in and out, and dehydration volume every hour. In any case, inform the responsible doctor as soon as possible.

The treatment group adopts the combined treatment mode of ECMO and CRRT, and the control group adopts the ECMO treatment mode. Any emergencies during treatment will be dealt with according to the expert consensus opinion of fulminant myocarditis.^[9,13]^ All nurses and doctors involved in the treatment are experienced professionals.

### 2.7 Observation indicators

1. Record the patient's basic vital signs changes (such as blood pressure, body temperature, heart rate, etc.), weaning time, hospital stay, etc.;
2. Monitor changes in laboratory indicators before and after treatment: such as N terminal pro B type natriuretic peptied, high-sensitivity troponin I, myocardial enzymes, oxygenation index (partial pressure of O_2_/fraction of inspiration O_2_), blood lactic acid (lac), liver and kidney function, coagulation function, arterial blood pH, etc.;
3. Echocardiographic indicators: left ventricular ejection fraction (LVEF), left ventricular end-diastolic diameter;
4. Complications and treatment outcomes: including weaning rate, in- and out-of-hospital mortality, complication rate (such as infection, bleeding, thrombosis, etc.).
5. Sequential Organ Failure Assessment (SOFA)^[14]^ score is used to assess the patient's organ condition.

### 2.8 Data collection and management

Observation indicators were collected before treatment, 24 hours, 48 hours, 72 hours, 96 hours after treatment, and 2 hours before weaning. At the same time, follow-up for 30 days to assess complications and treatment outcome. One or 2 assistants will collect and record the entire data. Personal information about potential participants and registered participants will be collected, shared and stored in an independent storage room to protect confidentiality before, during and after the test. The access to the database will be restricted to the researchers in this study team.

### 2.9 Statistical analysis

The SPSS 25.0 software was used for statistical analysis. Measurement data are expressed as mean ± standard deviation or median (quartile), and count data are expressed as number of cases. The independent sample *t* test or Mann–Whitney *U* test was used for the comparison of measurement data, the paired *t* test was used for the comparison within the group, and the Chi-Squared test or Fisher exact probability method was used for the comparison of count data. All are two-sided tests, with *P* < .05 as the difference is statistically significant.

## 3 Discussion

Patients with fulminant myocarditis progress rapidly and are often accompanied by difficult to correct hemodynamic disturbances or stubborn fatal arrhythmias. Therefore, the early use of effective mechanical assist devices is the key to improving the prognosis of such patients.^[3,15]^ It has been reported in the literature that the survival rate of mechanical assist devices for fulminant myocarditis is 57% to 80%.^[3,16]^ Early and timely ECMO placement to maintain effective circulatory perfusion is the key to reducing the mortality of FM. However, for long-term use of ECMO treatment, it should be noted that the probability of complications of ECMO treatment such as limb ischemia, abnormal coagulation function, bleeding, and infection will be greatly increased.^[17]^ Some researchers believe that ECMO combined with CRRT avoids the risk of bleeding at the puncture site during ECMO-assisted systemic anticoagulation. At the same time, it can measure the pressure of the ECMO membrane before and after the membrane, reduce the pressure measurement device, and reduce the risk of infection.^[18]^ Studies have shown that during EMCO treatment, about 50% of patients may be combined with CRRT treatment,^[19]^ which may be related to ECMO oxygenated blood flowing backward through the renal artery, ECMO-related inflammation activation, high anticoagulation state, hemolysis, etc. These factors are related.^[20]^ The integrated use of ECMO and CRRT in parallel can achieve joint support of multiple organs such as heart, kidney, and lung in 1 cardiopulmonary bypass pathway. However, some scholars believe that the use of CRRT during ECMO may increase the risk of in-hospital mortality.^[21]^ Therefore, whether the early application of CRRT is beneficial to the prognosis of patients with fulminant myocarditis still needs clinical research to verify.

This study also has the following limitations: the sample size included in this study is limited, which may affect the results. The planned follow-up time for this study is short, and there is no assessment of the long-term efficacy and safety of the treatment plan. If necessary, we will extend the follow-up time and pay more attention to its long-term efficacy.

## Author contributions

**Data curation:** Juan Wu, Hui Zhang.

**Funding acquisition:** Xihua Huang.

**Resources:** Hui Zhang, Yongxia Gao.

**Software:** Hui Zhang.

**Supervision:** Yongxia Gao, Xihua Huang.

**Writing – original draft:** Juan Wu, Hui Zhang.

**Writing – review & editing:** Juan Wu, Xihua Huang.
